# Molecular Genetic Features of Cerebral Cavernous Malformations (CCM) Patients: An Overall View from Genes to Endothelial Cells

**DOI:** 10.3390/cells10030704

**Published:** 2021-03-22

**Authors:** Giulia Riolo, Claudia Ricci, Stefania Battistini

**Affiliations:** Department of Medical, Surgical and Neurological Sciences, University of Siena, 53100 Siena, Italy; riolo@student.unisi.it (G.R.); claudia.ricci@unisi.it (C.R.)

**Keywords:** cerebral cavernous malformation, CCM, KRIT1, CCM2, PDCD10, protein structure, endothelial cells, signaling complex, microvessel lesions

## Abstract

Cerebral cavernous malformations (CCMs) are vascular lesions that affect predominantly microvasculature in the brain and spinal cord. CCM can occur either in sporadic or familial form, characterized by autosomal dominant inheritance and development of multiple lesions throughout the patient’s life. Three genes associated with CCM are known: *CCM1/KRIT1* (krev interaction trapped 1), *CCM2/MGC4607* (encoding a protein named malcavernin), and *CCM3/PDCD10* (programmed cell death 10). All the mutations identified in these genes cause a loss of function and compromise the protein functions needed for maintaining the vascular barrier integrity. Loss of function of CCM proteins causes molecular disorganization and dysfunction of endothelial adherens junctions. In this review, we provide an overall vision of the CCM pathology, starting with the genetic bases of the disease, describing the role of the proteins, until we reach the cellular level. Thus, we summarize the genetics of CCM, providing a description of CCM genes and mutation features, provided an updated knowledge of the CCM protein structure and function, and discuss the molecular mechanisms through which CCM proteins may act within endothelial cells, particularly in endothelial barrier maintenance/regulation and in cellular signaling.

## 1. Introduction

Cerebral cavernous malformation (CCM) is a disease predominantly affecting neurovasculature. These malformations consist of densely packed, enlarged capillary “caverns” generally arranged in a back-to-back pattern with no involvement of brain parenchyma [1]. Histologically, the lesions lack smooth muscle, elastic tissue, and all of the elements required for mature blood vessels. Clustered capillaries are lined by a single layer of endothelium included in a dense collagen matrix, and so characterized by an inhomogeneous vessel wall because of the presence of defective cell–cell junctions. This condition results in the impairment of the blood–brain barrier that predisposes it to episodes of thrombosis and bleeding. Due to the cerebral hemorrhages, patients may present an increased risk for stroke, seizures, motor and sensory deficits, and non-specific headaches [2,3]. However, many affected individuals are clinically asymptomatic during their entire life [4]. Apart from the brain and spinal cord location, CCMs are also seen in the retina, liver, kidney, and as hyperkeratotic cutaneous capillaryvenous malformations on the skin [3,5].

The penetrance has been calculated to be 0.5% in the general population worldwide (1 in 200–250 individuals), but the clinical prevalence is much lower because up to 50% of individuals with CCM are asymptomatic [6,7]. CCM-related clinical manifestations usually appear between the third and fifth decades of life, with no gender predominance, even if some cases have been also reported in children [8]. Clinical signs and symptoms are largely determined by the location, number, and size (from a few millimeters to several centimeters) of the lesions. Given that both size and the number of malformations can change over time, new signs and symptoms can arise at every stage of a patient’s life. The 3T Magnetic Resonance Imaging represents the gold standard for CCM detection [9]. Treatment differs between the administration of antiepileptic drugs in patients with seizures and surgical CCM resection in symptomatic patients, keeping in mind that lesions are not always easily accessible [6,10].

In order to devise new strategies to control and delay the disease progression, considerable efforts have been made to unravel both genetic and molecular causes, which are decisive in the onset of malformations. Here, we focus on gene–protein–disease mechanisms involved in the pathogenesis of CCMs, describing in detail how the identified genes drive the disease, which roles and functions are played by the product of each gene, what networks and signaling pathways are involved in which of these proteins, separately or together [11].

## 2. CCM Pathogenesis

Two forms of the disorder have been identified—sporadic (80%) and inherited (20%) disease. Sporadic cases, which occur in people with no family history of CCM, are mostly associated with the presence of a single lesion, while multiple lesions are frequently detected in familial CCM (fCCM) [12].

To date, both fCCM and sporadic forms have been attributed to mutations in three different genes, identified by linkage analysis and known as *CCM1/KRIT1*, *CCM2/MGC4607,* and *CCM3/PDCD10*.

The fCCM form has an autosomal dominant inheritance, with incomplete penetrance and variable expressivity [13]. The Knudsonian two-hit mechanism is the most accredited hypothesis to explain CCM pathogenesis [14]. According to this theory, loss of one allele due to a germline mutation in all cells (first hit) is followed by the occurrence, just in some cells, of a somatic mutation in the other allele (second hit), triggering the initiation of the lesions [12,15]. Animal experiments demonstrate that knockout mice for CCM genes die during embryogenesis because of heart deficiency and vascular defects, while mice carrying only one mutated allele rarely develop cavernous malformations [16]. These findings suggest that the loss of both copies of the gene represents the driving force for CCM pathogenesis.

Nevertheless, the definitive cause that determines CCMs remains an open question. In addition to this evidence, another important feature of the CCM condition is the heterogeneity of the phenotypes. More than 350 distinct *CCM1/CCM2/CCM3* mutations have been published to date [17], and patients carrying different mutations in different genes are clinically and phenotypically indistinguishable, although *CCM3* patients are often more severely affected with earlier symptomatic onset [18].

Of note, almost 5–15% of fCCMs do not show mutations in the three known CCM genes, raising the possibility of the existence of an additional gene correlated to the disease [19,20]. However, more than 15 years after the identification of the third CCM gene [19], this hypothesis is rather unlikely. It is more probable that the CCM families apparently negative for mutations in the three known CCM genes harbor a pathogenic variant not identified by the routinely used techniques [9].

All of the three discovered genes associated with CCMs found in mammals, vertebrates, and simple organisms (i.e., *Caenorhabditis elegans*), are well conserved among species, giving the advantage of exploiting numerous animal models to progress in the knowledge of this pathological condition [21].

### 2.1. CCM1 Gene

*CCM1/KRIT1* (OMIM#604214) was the first gene implicated in CCM to be identified on chromosome 7 locus q21.2. The coding region is represented by 16 of the 19 total exons that constitute the gene [22]. *KRIT1* is ubiquitously expressed in embryos with a pronounced rate in large vessels, while it progressively scales down in adulthood, becoming detectable predominantly in the nervous and epithelial tissues [23]. Knockout mice for this gene die at midgestation because of cardiac defects and vascular anomalies [24], showing the importance of *KRIT1* in vessel development and morphogenesis.

Mutations in this gene have been found in nearly all Hispanic American patients [25] and globally account for up to 50% of familial cases [26]. The clinical penetrance is estimated to be about 88% [1] and over 300 distinct mutations have been identified in this gene until now [17]. Frameshift, nonsense, missense, and splice site sequence variants are the most common mutations occurring in *KRIT1*, all resulting in early stop codons and, subsequently, in truncated and improperly working proteins. Moreover, rare insertions or deletions have also been identified [27,28,29,30,31,32].

### 2.2. CCM2 Gene

The second CCM locus, also known as *MGC4607* or *C7orf22* (OMIM#607929), identified on 7p13, consists of 10 coding exons [33]. Almost 20% of familial CCMs are due to mutations in this gene that was reported to be the only one with 100% of clinical and radiological penetrance [34]. However, recently, for the first time, a penetrance <100%, equivalent to 70%, has been reported for the *CCM2* gene [3]. More than 90 distinct mutations (missense, nonsense, frameshift, and splice site variants) have been found in the *CCM2* gene up till now [17].

As *KRIT1, CCM2* is ubiquitously expressed in the endothelium of various organs [35] and *CCM2*-silencing in zebrafish [36] and mice [37] revealed defects in arteries and veins formation, determining death during embryonal stages. Interestingly, mice heterozygous for *KRIT1* or *CCM2* do not develop lesions, unless the occurrence of a secondary hit, as reported by Shenkar et al. [38].

A lower number of lesions are commonly detected in *CCM2*-mutated patients in comparison to carriers of mutations in the other CCM genes, and the risk of an increase of the number of lesions during life is lower than in patients with mutations in the *KRIT1* gene [20]. In addition, hyperkeratotic cutaneous capillaryvenous malformations have been reported to be strongly associated with *KRIT1* variants, while patients with mutations in the *CCM2* gene are less prone to develop cutaneous lesions [39].

### 2.3. CCM3 Gene

*CCM3*, also named *PDCD10* (programmed cell death 10) (OMIM#609118), is the most recently discovered gene associated with CCM, identified by Bergametti et al. in 2005 after screening of patients with no mutations in *KRIT1* or *CCM2* [19]. *PDCD10* is a highly conserved pro-apoptotic gene, located on 3q26.1, containing seven coding and three non-coding exons. As reported for the other two genes associated with CCM disease, all the over 70 mutations identified in *PDCD10* result in protein loss of function [17]. Patients carrying mutations in the *PDCD10* gene are less common than *KRIT1* or *CCM2* carriers; only 10% of all familial CCM cases are due to mutations in this gene, with a penetrance of about 63% [40]. However, the clinical phenotype of patients with *PDCD10* mutations is often significantly more severe and symptoms onset occurs earlier in life [18]. *PDCD10* deletion in animal model causes aberrant vasculogenesis and hematopoiesis [41].

### 2.4. Any Other Additional Genes?

As previously reported, patients with no mutations in *KRIT1*, *CCM2*, and *PDCD10* genes might carry pathogenic variants in different genetic loci. Thus, the existence of an additional gene associated with CCM has been postulated. In addition to the three known genes, in 2008, Gianfrancesco et al. [42] proposed the zona pellucida-like domain containing 1 (*ZPLD1*) gene (3q12.3) as a new possible candidate implicated in CCM disease. However, further studies described this as a rare condition because the *ZPLD1* gene does not directly cause CCM, but may be implicated in some unidentified regulatory pathway associated with the disease [22,43]. Genetic screening currently includes the analysis of coding exons and exon/intron junctions followed by sequencing and deletion/duplication testing of *KRIT1*, *CCM2*, and *PDCD10*. Investigation on new genes responsible for CCM pathology is still ongoing, but it seems to be more likely that the familial cases negative for mutations in the three genes actually carry a pathogenic variant not identified by the commonly used diagnostic methods (e.g., a variant outside the screened exonic regions, deep intron variants, or a copy number neutral genomic rearrangement in one of the three genes) [9]. 

Key information of the three CCM genes is summarized in Table 1.

## 3. CCM Proteins

The inherited nature of CCM and the discovery of disease-related genes sparked interest in investigating their functional roles. The ubiquitous expression of the three CCM genes in various cells and tissues provides evidence for their contribution in several physiological and pathological conditions [44]. However, the cellular type most strongly associated with cavernous malformations is the endothelial cell. The role played by CCM genes in other cellular models strictly associated with lesions localization (i.e., neuronal cells, astrocytes, pericytes, and smooth muscle cells) is unknown, and there is no evidence about their direct contributions to CCM pathology [45]. There is probably a tight interplay between endothelial cells and the glia in the central nervous system (CNS), which may explain why mutations in CCM-related genes are followed by the appearance of lesions predominantly localized in the brain and spinal cord [46].

*KRIT1, CCM2*, and *PDCD10* genes encode for proteins different in structure and not sharing sequence homology. Each gene product is a multi-domain adaptor protein, which interacts with numerous binding molecules and participates in several different signaling pathways. On the other hand, KRIT1, CCM2, and PDCD10 proteins can exist into a heterotrimeric complex, the CCM signaling complex (CSC), being partners in numerous cellular processes that significantly affect CCM development and pathogenesis [47]. Thus, CCM proteins fulfill critical roles in many cellular events, such as cell polarity, cytoskeletal reorganization, cell proliferation, cellular adhesion, and migration, impacting angiogenesis, cell–cell junction integrity, vascular permeability, and apoptosis, whether as part of the ternary complex or not.

In the present review, each CCM protein and its related functions are described in a specific paragraph, while the converging molecular pathways and the role of the trimeric CCM complex are depicted in the last section.

### 3.1. KRIT1 Protein

**Figure 1:**
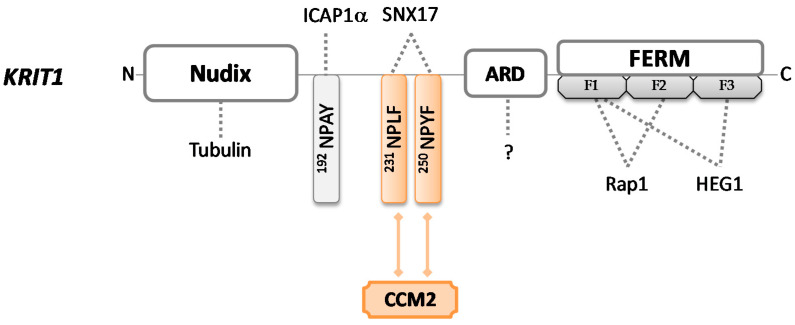
Schematic representation of KRIT1 protein. KRIT1 consists of a Nudix domain, three NPxY/F sites, four ankyrin repeat domains, and a FERM domain. FERM contains three subdomains (F1, F2, and F3) and each of them folds differently—F1 adopts a ubiquitin-like fold, F2 adopts an acyl–CoA binding protein fold, and F3 adopts a PTB domain-like fold. Colored forms and lines in the figure indicate domains and interactions required to bind CCM2 in order to build the CCM trimeric complex. Dotted lines indicate intermolecular interactions that may occur through each protein domain.

*KRIT1* gene encodes for the largest (84 kDa) and best-characterized of the three CCM proteins; named Krev interaction trapped protein 1 (UniProt #O00522), it is a 736-amino acid sequence without catalytic activity that can be found in many different subcellular sites. Several insights about KRIT1 functions have been obtained carrying out an extensive analysis of the structural motifs contained in the protein. KRIT1 consists of an *N*-terminal NUDIX domain, followed by three NPxY/F (Asn-Pro-X-Tyr/Phe) motifs, a central ankyrin repeat region (ARD), and a *C*-terminal FERM domain (band 4.1 ezrin radixin moesin domain) [48,49] (Figure 1).

The FERM module consists of three subdomains (F1, F2, and F3). In 1997, Serebriiskii et al. [50] firstly identified KRIT1 as a binding partner for Rap1, a member of the RAS family of GTPases, which plays important roles in both intercellular and cell–extra-cellular matrix (ECM) adhesion, as well as in cell proliferation pathways. Later, in 2012, it was demonstrated that the binding occurs via KRIT1 FERM domain [51] through F1 and F2 subdomains, while the F1–F3 interface forms a hydrophobic pocket that may interact with the cytoplasmic tail of the transmembrane heart of glass (HEG1) protein [52]. HEG1 is critical for the proper localization of KRIT1 at endothelial cell–cell junctions, contributing to junctional stability and participating in signaling due to the Rap1–KRIT1 complex [53]. KRIT1 mutants that cannot recruit Rap1 or HEG1 are associated with cardiovascular defects [52], suggesting that the set of these interactions plays an important role in the stabilization of cell–cell junctions and may account for KRIT1 involvement in CCM pathogenesis [54].

The three NPxY/F motifs mediate the interactions with other binding partners. The unique NPxY module interacts with the phosphotyrosine-binding (PTB) domain of a protein known as α-isoform of integrin cytoplasmic-associated protein-1 (ICAP1α), which mainly works as a suppressor of β1 integrin. ICAP1α competes with the integrin activators talin and kindlin, in order to modulate integrin activity and focal adhesion turnover. If talin and kindlin recruit actin bundles and favor cell–ECM attachment, ICAP1α inhibits the integrin–actin axis, disrupting cell adhesion and migration [55]. KRIT1 acts as a competitive inhibitor for the interaction between ICAP1α and β1 integrin. Thus, because ICAP1α utilizes the same PTB site to bind KRIT1 and the cytoplasmic portion of β1 integrin when it is bound to KRIT1, it cannot interact with, and consequently, negatively regulate the integrin activity, resulting in increased integrin-mediated cell adhesion and hyperangiogenesis [35,55]. Furthermore, ICAP1α exhibits a stabilizing function toward KRIT1 and induces its translocation into the nucleus, preventing proteasomal degradation. This evidence highlights how KRIT1 can fulfill such a dual role (cytoplasmic and nuclear), but its exact function in the nucleus remains at present completely unknown [56]. Of note, KRIT1 may provide potential crosstalk between integrin and Rap1 signaling by communicating in concert with ICAP1α and Rap1, also suggesting the involvement of the latter protein in integrin-mediated adhesive events. Importantly, mutations in the KRIT1 gene may nullify this molecular association, confirming the crucial implication of KRIT1 in cell–cell and cell-matrix adhesion [57,58].

Through the NPxF motifs, KRIT1 recruits different binding proteins, including CCM2. KRIT1 and CCM2 show similar spatiotemporal expression patterns, demonstrating that they are strictly interconnected [59]. It is worth noting that CCM2 binding to KRIT1 does not abrogate KRIT1–ICAP1α interaction, supporting the hypothesis that a ternary ICAP1α/KRIT1/CCM2 complex may exist. In 2005, Zawistowski et al. [60] reported that the subcellular localization of KRIT1 is differentially influenced by these two proteins—if ICAP1α induces nuclear translocation, CCM2 maintains KRIT1 into the cytosol. However, it has been recently suggested that the whole complex ICAP1α/KRIT1/CCM2 shuttles back and forth from cytoplasm to nucleus and plays different roles in different cellular compartments [45].

KRIT1 uses NPxF motifs also to recruit sorting nexin 17 (SNX17) and localize into intracellular vesicles, but the effects produced by this interaction remain to be elucidated [61].

Furthermore, the NPxY/F region supports changes in protein shape, which in turn define the functional outputs. In fact, KRIT1 functions depend on the conformation that the protein acquires in subcellular locations—an open conformation enables the KRIT1-ICAPα interaction [62], while a closed conformation mainly exposes the NUDIX region [63].

The *N*-terminal NUDIX domain, recently described as pseudo-NUDIX because it is lacking hydrolase activity normally showed by NUDIX domains, is responsible for the binding of KRIT1 to tubulin, making KRIT1 a microtubule-associated protein. Therefore, KRIT1 modulates the cytoskeletal organization of endothelial cells, playing a crucial role also in cell morphology. Gunel et al. [10] pointed out that the loss of KRIT1 results in impaired tubulogenesis and consequently leads to abnormal vessel development, which is typical of the CCM lesions.

Finally, four ankyrin repeat domains are functionally available for the interaction with several molecules of different origins and natures [64]. Their precise roles in CCM pathogenesis remain elusive, but interestingly, the combination of ankyrin repeats with the FERM domain is apparently unique to KRIT1 [58], suggesting that it is probable that further functions of this protein have yet to be discovered. 

Collectively, KRIT1 regulates several cellular processes and plays numerous pivotal roles in different signaling pathways when assembled with CCM2 and PDCD10 into the CSC.

### 3.2. CCM2 Protein

**Figure 2:**
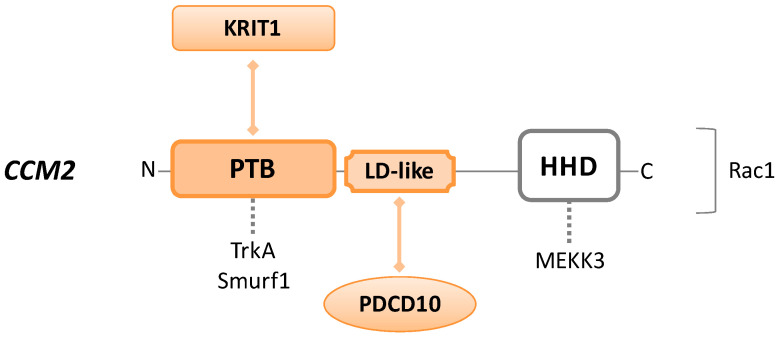
Schematic representation of CCM2/malcavernin protein. CCM2 consists of an *N*-terminal phosphotyrosine-binding (PTB) domain, a *C*-terminal harmonin homology domain (HHD), and a central LD-like motif. Colored forms and lines in the figure indicate domains and interactions required to build the CCM trimeric complex. Dotted lines indicate intermolecular interactions that may occur through each protein domain. Rac1 is placed on the right of the CCM2 sequence because the precise binding site is unknown.

The *CCM2* gene encodes for a 444-amino acid product referred to as CCM2 or malcavernin (48 kDa) (UniProt #Q9BSQ5), a scaffolding protein without any enzymatic activity. Structurally, from *N*-terminus to *C*-terminus, malcavernin consists of a PTB domain, an LD-like motif, and a helical harmonin homology domain (HHD) (Figure 2).

Malcavernin is central to the CCM trimeric complex organization, as the PTB and LD-like motif interact with NPxF of KRIT1 and focal adhesion targeting homology (FAT-H) domain of PDCD10, respectively. In this manner, CCM2 works as a linker, bringing together KRIT1 and PDCD10, which otherwise have no affinity for each other [2]. Loss of function mutations in any of these binding domains disrupt the CSC, alter the integrity at cell–cell junctions, and cause aberrant cell–ECM adhesion [65].

However, KRIT1 and CCM2 are more strictly connected to each other than to PDCD10. As mentioned before, KRIT1–CCM2 interaction is essential for CCM2 localization at the cell–cell junctions, and for the maintenance of KRIT1 into the cytoplasm. Interestingly, CCM2 depletion leads to the loss of KRIT1 from the cell membrane, inducing endothelial barrier dysfunction [66]. Moreover, several studies describe the crucial involvement of CCM2 in the KRIT1–HEG1 pathway, required for normal cardiovascular development [54,67,68].

The CCM2–PTB domain is also able to bind Tropomyosin receptor kinase A (TrkA), known to be involved in apoptosis, and Smad ubiquitin regulatory factor 1 (Smurf1), an upstream RhoA signaling protein [69].

Among other functions, CCM2 can recruit Rac1 protein, interfering with the actin cytoskeleton, or bind to MEKK3 (mitogen-activated protein kinase (MAPK) kinase kinase 3) via the HHD region, affecting its signaling cascade. MEKK3 changes cell morphology in response to the hyperosmotic shock and regulates endothelial to mesenchymal transition (EndMT), a condition that shows characteristics frequently seen in CCM lesions (loss of integrity in cell–cell junctions and increased cell migration and proliferation activity) [70]. 

The aforementioned interactions of CCM2 with Smurf1, Rac1, and MEKK3 affect signaling cascades that are also influenced by KRIT1 or PDCD10, but it is still not well characterized whether CCM proteins interfere with the pathways as part of the CSC or not. These converging molecular activities will be deeply explained later.

Recently, Zheng et al. [71] identified a paralog of malcavernin, termed CCM2 protein-like (CCM2L), the sequence of which is highly similar, but not entirely homologous, to CCM2 and which displays some features different from CCM2. First of all, the expression of CCM2L is restricted to endothelial cells, in contrast to CCM2, which is ubiquitously expressed. As CCM2L lacks an LD-like motif for binding to PDCD10, it directly competes with CCM2 for binding to KRIT1 but cannot work as the hub of CSC, and consequently inhibits the activities mediated by the KRIT1/CCM2/PDCD10 trimeric complex. However, in some cases, CCM2L can compensate for many functions of malcavernin. For example, it can inhibit the nuclear translocation of KRIT1, maintaining the protein into the cytosol, induce cell death by activating TrkA via the C-term PTB domain, and work as an osmosensing scaffold protein (OSM) for the MEKK3 pathway [72]. Even though the exact functions of CCM2L are still poorly defined, it is clear that this CCM2 homolog has some roles complementary to malcavernin in CCM pathogenesis.

### 3.3. CCM3 Protein

**Figure 3:**
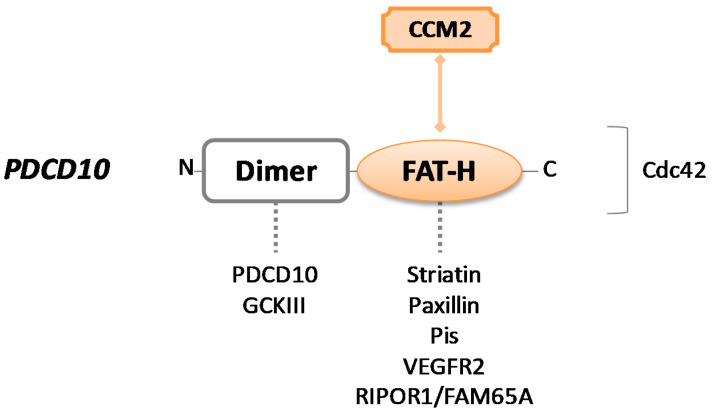
Schematic representation of CCM3/PDCD10 protein. PDCD10 consists of a dimer site and a focal adhesion targeting homology (FAT–H) domain. Colored forms and lines in the figure indicate the crucial interaction with CCM2 to build the CCM trimeric complex. Dotted lines indicate intermolecular interactions that may occur through each protein domain. The binding site for the Cdc42 protein is unknown.

The *PDCD10* gene encodes for PDCD10, a protein also named TF-1 cell apoptosis-related protein (TFAR15) (UniProt #Q9BUL8). It is a 25kDa protein consisting of 212 amino acids and composed of two main domains: An *N*-terminal dimerization domain and a focal adhesion targeting homology (FAT–H) domain, localized at the *C*-terminus of the protein [73] (Figure 3).

The hydrophobic patch 1 (HP1) region of α-helical FAT–H domain is responsible for the crucial interaction between PDCD10 and the LD motif of CCM2 for the assembly of CSC [74]. Although the interaction between CCM2 and PDCD10 has stabilizing effects for all the proteins of the complex, protecting them from degradation, PDCD10 predominantly operates outside of the CCM signaling complex.

The best-known function singularly mediated by PDCD10 involves its dimerization domain, which consists of four α-helices and alternatively supports homodimerization (with PDCD10 itself) and heterodimerization with the striatin interacting phosphatase and kinase (STRIPAK) complex. STRIPAK components are represented by striatins and striatin-interacting proteins STRIP1/2, protein phosphatase 2A (PP2A), protein kinases belonging to the germinal center kinase III (GCKIII) family (i.e., STK25, STK24, and MST4), etc. [75,76,77]. 

The STRIPAK complex is a central regulator node of several processes in endothelial cells. First of all, it is crucial for Golgi assembly and positioning during cell migration, and PDCD10 has been shown to actively participate in this process, stabilizing the GCKIII kinases through anchorage to GM130 cisGolgi-resident protein and promoting proper Golgi orientation [78]. Of note, the loss of PDCD10 downregulates STK25 activity, leading to the aberrant repositioning of both Golgi apparatus and centrosome toward the leading edge of the cell, consequently disrupting cellular directional migration [79]. Then, as a STRIPAK component, PDCD10 plays a crucial role as a dual regulator of exocytosis. It can recruit STK24, which is known as an exocytosis inhibitor, neutralizing it to enhance exocytosis, while PDCD10-UNC13 interaction has the opposite effect [80]. This regulatory function of PDCD10 is essential in endothelial cells where excessive secretion of angiopoietin-2 (ANGPT-2) to the extracellular space strongly impacts junctional and vascular stability [81,82]. Lastly, the MST4 kinase of the GCKIII family interacts with PDCD10 under conditions of oxidative stress to regulate cell polarity and the NF–kB signaling pathway [83]. 

Using the FAT–H domain, PDCD10 may form other dynamic interactions apart from CCM trimeric complex. It can recruit striatins and paxillin or bind to phosphotidylinositides (PIs) that facilitate its translocation from the Golgi to the plasma membrane [84]. Through the same binding region, PDCD10 contributes to angiogenesis and cell proliferation interacting with VEGFR2—it has been described that PDCD10 mutants show a reduced VEGFR2 expression because of pronounced endocytosis, resulting in aberrant vascular development and cell death [41]. Recently, it was reported that PDCD10–FAT–H can also directly bind to Rho family interacting cell polarization regulator 1 (RIPOR1), participating in cellular morphology and migration via RhoA signaling [85]. 

PDCD10, as its name suggests, possesses pro-apoptotic activity. As already described, CCM2 is involved in apoptosis through the binding to TrkA and, most of the time, this interaction happens simultaneously to PDCD10–STK25 association, forming a large complex involved in apoptosis [86]. However, PDCD10 can lead to apoptosis through an alternative mechanism, by activation of the caspase-3 pathway [87]. In addition, pro-survival effects have also been reported for this protein [88]. 

Moreover, PDCD10 importantly contributes to cellular proliferation. Interestingly, overexpression of PDCD10 in CCM2-deficient cells restore the cells’ ability to proliferate, while upregulation of CCM2 in PDCD10-defective cells does not rescue proliferative activity [74]. PDCD10 loss also results in defective cell migration, due to suppression of RhoA signaling that dysregulates cytoskeleton organization [11].

## 4. CCM Signaling Complex

CCM proteins singularly operate and affect different signaling pathways, but they are also involved in converging molecular signal cascades, whether acting as a trimeric complex or not.

### 4.1. Signaling Pathways Modulated by the Rho Family of GTPases

**Figure 4:**
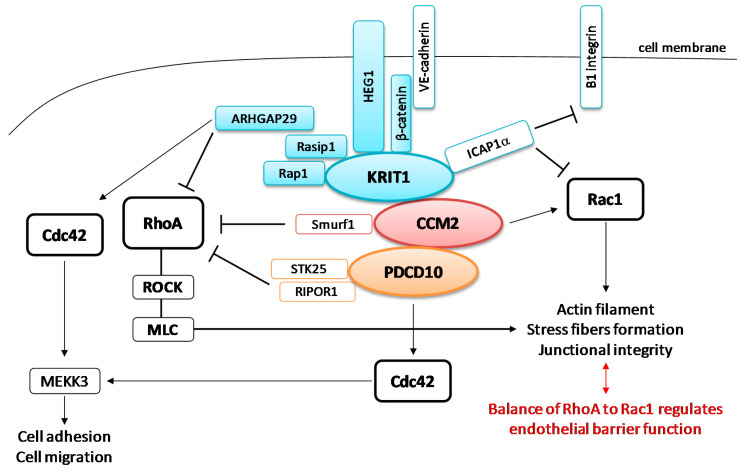
CCM signaling complex (CSC) complex involved in Rho GTPase signaling. KRIT1 anchors to the cell membrane through β-catenin and HEG1, and then associates with Rap1 and Rasip1, which recruits ARHGAP29, forming junctional signaling involved in the regulation of RhoA and Cdc42 pathways. All components of the junctional signaling are indicated in blue. CCM2 associates with Smurf1 to inhibit RhoA signaling. PDCD10 may control RhoA via interactions to RIPOR1 or STK25. All the three CCM proteins negatively regulate RhoA-ROCK (Rho-associated coiled coil-forming kinase) blocking myosin light chain (MLC) phosphorylation and preventing excessive stress fiber formation and endothelial barrier dysfunction, which contribute to the dysmorphic vessels that typify CCM lesions. ARHGAP29 and PDCD10 in the STRIPAK complex are involved in the regulation of Cdc42 through mechanisms not well understood, as well as is unclear how Cdc42 finely regulates MEKK3 pathways.

CCMs are characterized by a leaky vasculature deriving from altered cell–cell junctions. In the endothelium, cell–cell and cell–ECM adhesions mainly occur through transmembrane vascular endothelial (VE)-cadherins and integrins, respectively. Crosstalk between cadherins and integrins creates a complex signaling network that is crucial for normal endothelial tissue development. Several kinases and receptors are involved in this network, including the Rho family of GTPases (RhoA, Cdc42, and Rac) and CCM proteins, which strictly regulate endothelial barrier maintenance, cell polarity, and cytoskeletal contractility (Figure 4).

RhoA signaling is one of the best-characterized signaling cascades controlled by CCM proteins. Several studies have reported that it importantly contributes to CCM disease since vascular permeability and stress fiber formation are enhanced in cases of CCM gene silencing [66,89,90].

RhoA and its effector, Rho-associated coiled coil-forming kinase (ROCK), are involved in remodeling the actin cytoskeleton and junctional integrity, in order to enable endothelial cells to properly migrate. CCM proteins are essential for inhibiting RhoA accumulation and preventing the hyperactivity of Rho kinase and endothelial barrier dysfunction.

KRIT1 directly associates with β-catenin, HEG1, Rap1, and Ras effector protein 1 (Rasip1) in the junctional signaling, forming a larger complex for the stabilization of endothelial junctions [45]. This helps turn off RhoA activity, preventing downstream myosin light chain (MLC) phosphorylation by ROCK and excessive stress fiber formation, which has been linked to weakened junctions between cells and increased leakage from blood vessels. CCM2 promotes RhoA degradation through binding to Smurf1 [91,92], while PDCD10 acts through either STK25 of GCKIII kinases or RIPOR1 protein to regulate the same actin-remodeling pathway [70]. In particular, Stockton et al. [66] reported that physical interaction between KRIT1 and CCM2 is required for a strong suppression of RhoA signaling, while PDCD10 may participate individually or in complex with the other two CCM proteins. Since KRIT1/CCM2/PDCD10 dysfunction promotes abnormal RhoA–ROCK signaling, inhibitors of this biochemical cascade have been considered as a therapeutic strategy to treat CCMs patients [93].

Endothelial motility and morphology during angiogenesis are regulated by Rac1 activity [94]. Both KRIT1 and CCM2 modulate Rac1 signaling—KRIT1–ICAP1α inhibits Rac1, whereas CCM2 colocalizes with Rac1 at membrane ruffles in endothelial cells, promoting the endothelial barrier and participating in actin remodeling [95]. 

In 2014, Van den Berg et al. [55] suggested that endothelial barrier function can be achieved through a fine balance between RhoA and Rac1 expression—low activity of RhoA and high activity of Rac1 improve the junctional integrity of the endothelium, whereas the opposite condition decreases the barrier function.

Although the precise molecular mechanism is still under study, it seems that CSC also promotes the activation of Cdc42, a downstream effector of Rap1 signaling, which appears to be essential in a variety of cellular processes, from cytoskeleton reorganization and junctional rearrangement [96] to cell polarity, focal adhesion formation, and migration [97]. Mutations disrupting the trimeric complex drastically downregulate the Cdc42 cascade, causing defective angiogenesis and actin filament disassembly. More interestingly, mutated Cdc42 reproduces the phenotype of CCM with no changes in CCM proteins level [98]. In addition, PDCD10 may also interact with the Cdc42 as part of the STRIPAK complex, independently from CSC, confirming the fundamental role of this Rho GTPase in CCM lesions [99].

### 4.2. MEKK3 Downstream Signaling Pathways

In addition to the role of Rho GTPases in CCM disease, another well-known pathway is represented by the MEKK3 signaling cascade, which is implicated in cell migration and proliferation, and required for proper vessel development [100]. 

**Figure 5:**
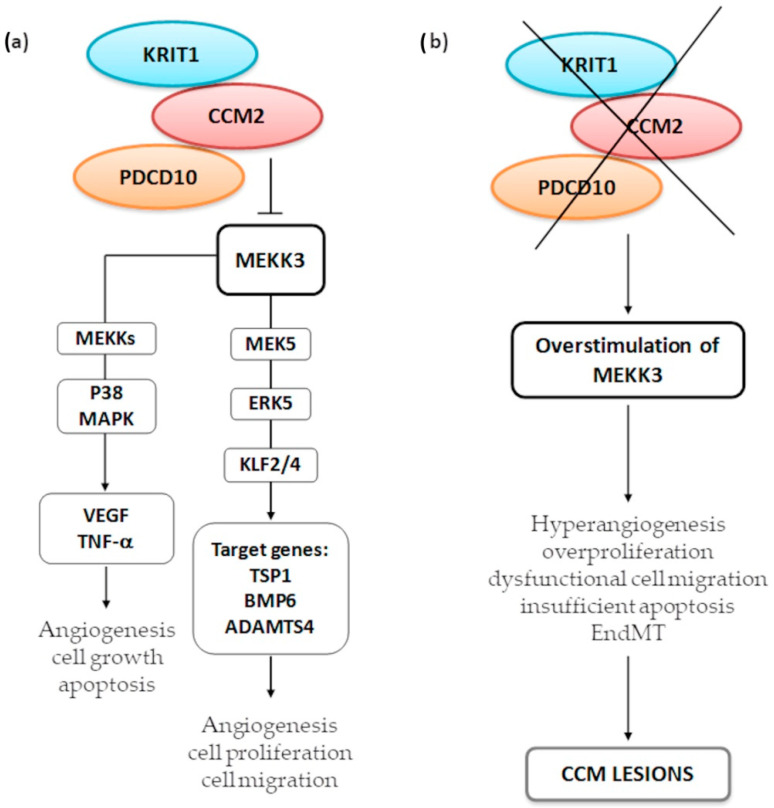
MEKK3 signaling regulated by CCM2 in the CSC complex (**a**) and disrupted signaling due to CCM protein depletion (**b**). CCM2 binds to and inhibits MEKK3, preventing its ability to phosphorylate downstream kinases. MEKK3-MEK5-ERK5-KLF2/4 is an underlying signaling cascade for angiogenesis, ensuring cell–cell junctional integrity and promoting endothelial barrier. Conversely, p38 mitogen-activated protein kinase (MAPK) activation mediated by MEKK3 is required to control pro-survival and pro-apoptotic stimuli. CCM proteins strictly control both signaling cascades, in order to avoid MEKK3 hyperactivation, which may result in excessive cell proliferation, dysfunctional migration, EndMT, and altered response to apoptosis, all features characterizing CCM lesions.

MEKK3 is an upstream kinase belonging to both the MEKK3-MEK5-ERK5-KLF2/4 and the p38 mitogen-activated protein kinase (MAPK) signaling pathways (Figure 5).

Several studies have reported that CCM deficiency results in hyperstimulated MEKK3 [72,101,102,103], which elicits activation of the downstream effectors Kruppel-like factor 2/4 (KLF2 and KLF4), transcription factors commonly upregulated in inflammation and during hemorrhages [100].

KRIT1–CCM2 complex sequesters MEKK3, which in turn activates MEK5 and then ERK5, providing proper expression of KLF2/4 to ensure cell–cell junctional integrity and adequate endothelial permeability. Impaired MEKK3 signaling induces defective angiogenesis [104] and overexpressed KLF2/4, which are pivotal players in the initiation of the CCM pathogenesis [105]. These transcription factors increase cell proliferation and dysfunctional migration and suppress the expression of several target genes: the inhibitor of angiogenesis thrombospondin 1 (TSP1), the bone morphogenetic protein 6 (BMP6), which drives EndMT, and a disintegrin and metalloproteinase with thrombospondin motifs 4 (ADAMTS4), involved in cardiac development [102,103].

Upregulation of MEKK3 may also trigger the MEKK3–p38 MAPK signaling pathway, an important mediator of VEGF activation and TNF-induced apoptosis [106,107]. 

What exact role is played by CCM2 in the regulation of p38 MAPK is still unclear; however, Plummer et al. [16] reported that CCM2 silencing may influence downstream p38 signaling, which is critical for the formation of new vessels and the maintenance of existing vessel architecture.

### 4.3. Other Signaling Cascades

The CSC unfolding gives rise to the dismantling of junctions and the mislocalization of VE-cadherin. This results in the β-catenin release from the cell membrane and translocation into the nucleus, where it promotes gene expression and induces aberrant vessel wall and lumen formation [108]. 

BMP6 is one of the transcription factors upregulated by this cascade, which in turn activates the transforming growth factor-β (TGF-β), having a role in EndMT.

In 2010, Wustehube et al. [109] found that KRIT1 overexpression upregulates HEY1 and DLL4, two major players in Notch signaling, providing a proper vascular development. Conversely, KRIT1 silencing entails a reduced activity of the same pathway, which may generate vascular defects and contribute to the pathogenesis of CCMs. Notch inhibition induced by KRIT1 silencing also upregulates BMP6, suggesting that TGFβ and Notch pathways are not simply parallel, but probably converge in promoting the initiation of EndMT [110].

PDCD10, similarly to KRIT1, affects Notch signaling. PDCD10 downregulation results in upregulated BMP6 and ERK1/2 transcriptional activity, triggering EndMT and hyperangiogenesis [110,111,112], respectively.

Disorganized VE-cadherins can additionally disrupt the cellular response to oxidative stress, leading to increased intracellular reactive oxygen species (ROS). Thus, as previously reported, the absence of KRIT1 corresponds to decreased expression of SOD2, and therefore increasing oxidative damage in the cell [77,113]. PDCD10 deletion produces the same effect, suggesting that CSC may be a determinant factor in cell survival after oxidative stress stimulation [78]. 

**Figure 6:**
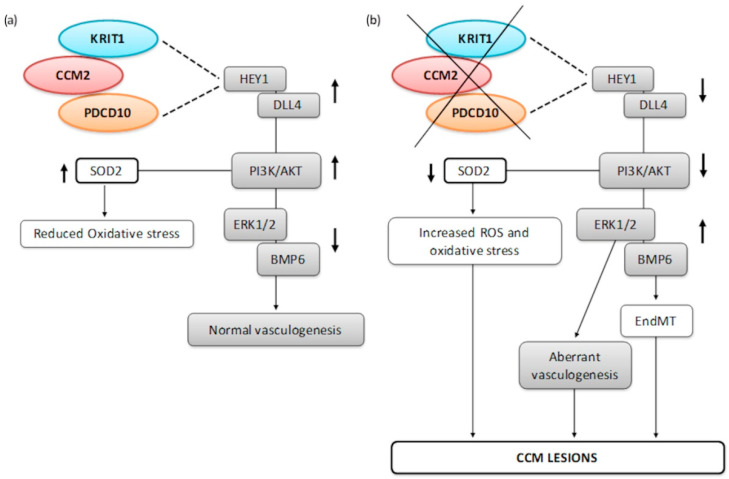
Other key signaling pathways in CCM pathogenesis: Notch Signaling and response to oxidative stress (**a**) compared to altered cascades in the absence of CSC (**b**).

A schematic representation of the aforementioned signaling involving CCM proteins is depicted in Figure 6.

Notch signaling (depicted as grey rectangles) increases HEY1 and DLL4, which in turn increase PI3K/Akt. Then, active Akt (Protein Kinase B) dephosphorylates ERK1/2 ensuring proper vasculogenesis. KRIT1 and PDCD10 directly participate in Notch signaling and depletion of both proteins importantly impact this pathway, resulting in aberrant vessel formations and EndMT, because of BMP6 deregulation. The AKT downstream factors of the Notch cascade also control SOD2 expression, reducing reactive oxygen species (ROS) accumulation and oxidative stress inside the cells. Loss of KRIT1 is predominantly associated with enhanced intracellular ROS levels.

In addition, also autophagy is related to CCM formation [114]. It has been reported that KRIT1/CCM2/PDCD10-mutated endothelial cells are characterized by inadequate autophagy mechanisms, which determine the progression of the disease. This evidence demonstrates that CCM proteins, singularly or in a complex, have a protective role in endothelial cells, preventing vascular dysfunction and aberrant cellular differentiation.

## 5. Conclusions

Cerebral cavernous malformation is recognized as a genetic disease due to loss of function mutations occurring in one of the three CCM-related genes, known as *KRIT1*, *CCM2*, and *PDCD10*. CCM proteins are binding partners of different molecules, scaffolding proteins, and kinases, through which they regulate numerous cellular processes. Despite their role as single effectors, KRIT1, CCM2, and PDCD10 can also associate with each other, forming a trimeric complex (CSC) involved in a larger network, in which several signaling pathways converge.

Collectively, CCM proteins are involved in vascular development and maintenance of blood vessel architecture, in addition to contributing to cell–cell junctional stability, participating in cell–ECM adhesive events, and regulating cytoskeletal reorganization, cell morphology, and migration. Mutations in one of the three CCM genes lead to loss of function of proteins, also impairing the function of the complex, resulting in endothelial barrier dysfunction and hyperpermeable blood vessels, which are prone to leakage and bleeding.

However, the landscape of interactions and signaling pathways controlled by the CCM proteins, whether as trimeric compounds or as single effectors, is very complex, and many questions remain unanswered.

First, *KRIT1*, *CCM2*, and *PDCD10* genes are ubiquitously expressed in cells and tissues, but it is not well established why mutations in CCM genes predominantly affect endothelial cells of blood vessels in the brain and spinal cord. Thus, further studies are required to unravel a potential interplay between endothelial and neuronal cells in the formation of CCM lesions.

Second, among the large number of cellular functions that have been attributed to the CCM proteins, some roles of KRIT1, CCM2, and PDCD10 in endothelial cells remain controversial. The interactions and/or the impaired signaling pathways that trigger CCMs formation need to be elucidated. A better understanding of CCM proteins in underlying disease mechanisms may shed light on druggable biochemical pathways that can be targeted by new therapeutic strategies.

Finally, several efforts are still required to define some pharmacological approach that may prevent CCMs from becoming symptomatic or arrest the progression of the disease.

## Figures and Tables

**Table 1:** Summary of cerebral cavernous malformation (CCM) genes and relative proteins. The number of identified mutations is referred to data included in the Human Gene Mutation Database (HGMD) [32].

Gene.	OMIM	Genetic Locus	Protein	UniProt	Identified Mutations
*CCM1/KRIT1*	#604214	7q21.2	KRIT1	#O00522	>300
*CCM2/MGC4607*	#607929	7p13	Malcavernin	#Q9BSQ5	>90
*CCM3/PDCD10*	#609118	3q26.1	PDCD10/TFAR15	#Q9BUL8	>70
